# Biological Effect of Audible Sound Control on Mung Bean (*Vigna radiate*) Sprout

**DOI:** 10.1155/2014/931740

**Published:** 2014-08-07

**Authors:** W. Cai, H. He, S. Zhu, N. Wang

**Affiliations:** ^1^Ningbo Institute of Technology, Zhejiang University, 1 Qianhu South Road, Ningbo, Zhejiang 315100, China; ^2^Key Laboratory of Equipment and Informatization in Environment Controlled Agriculture, Zhejiang University, 866 Yuhangtang Road, Hangzhou, Zhejiang 310058, China; ^3^Department of Biosystems and Agricultural Engineering, Oklahoma State University, 111 Ag Hall, Stillwater, OK 74078, USA

## Abstract

Audible sound (20–20000 Hz) widely exists in natural world. However, the interaction between audible sound and the growth of plants is usually neglected in biophysics research. Not much effort has been put forth in studying the relation of plant and audible sound. In this work, the effect of audible sound on germination and growth of mung bean (*Vigna radiate*) was studied under laboratory condition. Audible sound ranging 1000–1500 Hz, 1500–2000 Hz, and 2000–2500 Hz and intensities [80 dB (A), 90 dB (A), 100 dB (A)] were used to stimulate mung bean for 72 hours. The growth of mung bean was evaluated in terms of mean germination time, total length, and total fresh weight. Experimental results indicated that the sound wave can reduce the germination period of mung bean and the mung bean under treatments of sound with intensity around 90 dB and frequency around 2000 Hz and significant increase in growth. Audible sound treatment can promote the growth of mung bean differently for distinct frequency and intensity. The study provides us with a way to understand the effects and rules of sound field on plant growth and a new way to improve the production of mung bean.

## 1. Introduction

There are scientific literatures related to studies on the effects of subjecting seeds and plants to sound waves or magnetic field [1–9]. The vast majority of these papers deal with ultrasonic (above 20000 Hz) or subsonic (below 20 Hz) frequencies studying effects at the cellular and genetic levels. Little has been done with audible frequencies (20–20000 Hz) on seeds or whole plants and what has been done is mostly with single frequencies [1, 10–14]. Early studies have shown that some frequencies affect seed germination and the growth of plants differently than others [12, 13, 15]. Recently, studies have been done on the use of music to improve crop yield and quality in plants such as tomato, vegetables, and barley [15, 16]. Hou et al. [17] used audible sound waves to stimulate more than 50 different crops and achieved remarkable effects.

Recent studies have revealed that audible sound stimulation has a great potential to improve plant growth and the quality of products. However, till now, the proper mechanism of sound effects on plant is unknown; it is necessary to establish the mechanism and to develop models for application of this potential technology as well as to do experiments to find the best “sound frequencies and intensities” for different kind of plants growth.

Mung bean (*Vigna radiate*) is a popular growing legume and is widely consumed in form of mung bean sprouts in Asian cuisine, and the growth cycle of mung bean is short, which enables us to conduct more repetitive experiments. Exploring a new way to grow mung bean can improve mung bean production and satisfy consumers' requirements. Therefore, the objective of this work was to find the biological effect of audible sound on the germination and growth of mung bean and to find the best combination sound frequency bands and intensities for mung bean growth.

## 2. Materials and Methods 

### 2.1. Characteristics of Audible Sound

**Figure 1:**
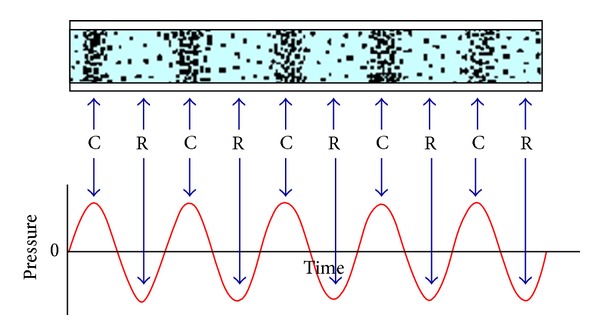
The identity between the longitudinal characterization of a sound wave in air and the pressure-time fluctuations.

Audible sound wave, like any other sound wave, is a mechanical wave that results from the back and forth vibration of the particles of a medium through which the sound wave is moving.  Figure 1 shows the identity between the longitudinal characterization of a sound wave in air and the pressure-time fluctuations that it creates at a fixed detector location. There are regions (known as compressions and rarefactions) in the air where the air particles are compressed together and other regions where the plant particles are spread apart as a result of the longitudinal motion of the air particles. The frequency of a sound wave refers to how often the particles of a medium vibrate when a wave passes through the medium and the intensity of a sound wave is a combination of its rate and density of energy transfer.

Similarly, if a sound wave is moving through a plant or plant seeds, then particles of plant or plant seeds will be displaced both rightward and leftward as the energy of the sound wave passes through them. The motion of the particles is parallel to the direction of the energy transport. We assume there are biological effects of audible sound field on plant growth and seed germination because of sound energy transport and the degree of the effect is dependent on sound frequency and sound intensity.

### 2.2. Seed Materials

Mung beans (*Vigna radiate*, production place: Hunan, China), with moisture content of 11%, were used in the experiments. To prevent absorption of moisture, they were stored in a dry cabinet under 20°C until required.

### 2.3. Experimental Design for Sound Treatment

As shown in Table 1, experiments were conducted through three trials according to the processing sound with different swept frequency bands (SFB) (1000–1500 Hz, 1500–2000 Hz, and 2000–2500 Hz) plus background sound. For each trial, there were four groups: 80 dB treatment group, 90 dB treatment group, 100 dB treatment group, and control (quiet, the background sound pressure level (SPL) was below 55 dB) group. Each group included four symmetry cultivation units (namely, four repeats).

**Figure 2:**
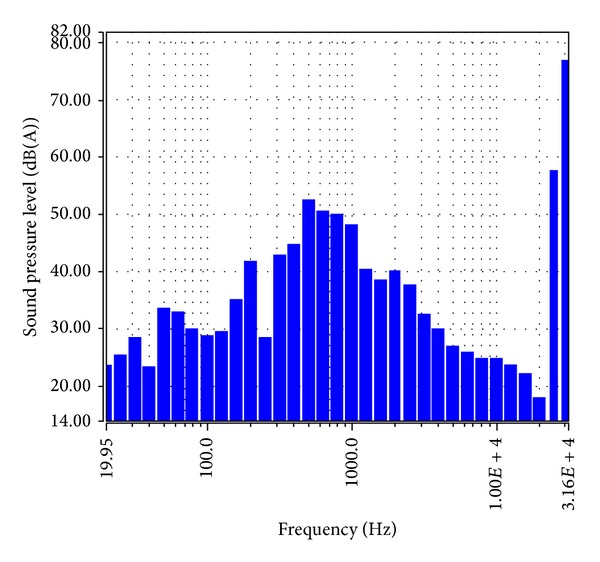
The octave analysis chart of background sound in the test room.

The environmental parameters of four groups that were maintained in the test room were almost the same. The octave analysis on background sound of the room is shown in Figure 2, and the light was supplied by white fluorescent lamps. The close environmental parameters of three groups can be achieved so that the growth difference between them only comes from the sound pressure level (SPL). The environmental parameters are as follows: temperature is 28 ± 2°C, humidity is 65 ± 6%, and illumination is 0.0622 ± 0.0027 *μ*E/m^2^/s (day) and 0.003 *μ*E/m^2^/s (night).

One hundred seeds of mung bean (about 68 mg for each seed with similar shape and size) were selected for every cultivation unit. Seeds were evenly sprinkled to the four symmetry cultivation units of the automatic machine of bean sprouts, respectively. Sound with relative swept frequency band was continuously played during every trial, and watering was done by the automatic machine for 3 minutes every half hour.

### 2.4. Germination Tests

The germination tests were performed in our experiments. A seed was considered a germinated seed if the embryo axes protruded more than 1 mm. Counts were made every three hours. Total number of seeds germinated was counted. Germination rate, the average number of hours required for seeds to germinate, was expressed as mean germination time (MGT). Mean germination time was calculated by the following equation:

(1) $$\begin{array}{c} \text{MGT}=\sum \frac{nc}{N}, \end{array}$$

where* n *is the number of seeds newly germinated (just germinated criterion) at time* c*,* c* is the germination time of seeds newly germinated ( = 3,6, 9,…), and *N* is the total number of seeds germinated.

### 2.5. Growth Tests

The growth tests were carried out in the experiments. At the 72nd hour, the total stem length, total root length, and total fresh weight were measured.

The growth of mung bean was evaluated in terms of total stem length and total root length in cm and total fresh weight in g.

### 2.6. Statistical Analysis

There is only one factor difference in each trial, and we only investigate one factor (SFB or SBL); therefore, data were analyzed using an analysis of variance (ANOVA) multiple comparison (single factor). The differences between the growth parameters of mung bean treated by audible sound and control were tested by the method. Treatment effects were considered to be significant at *P* < 0.05.

## 3. Results

### 3.1. Audible Sound Effect on the Germination of Mung Bean

**Figure 3:**
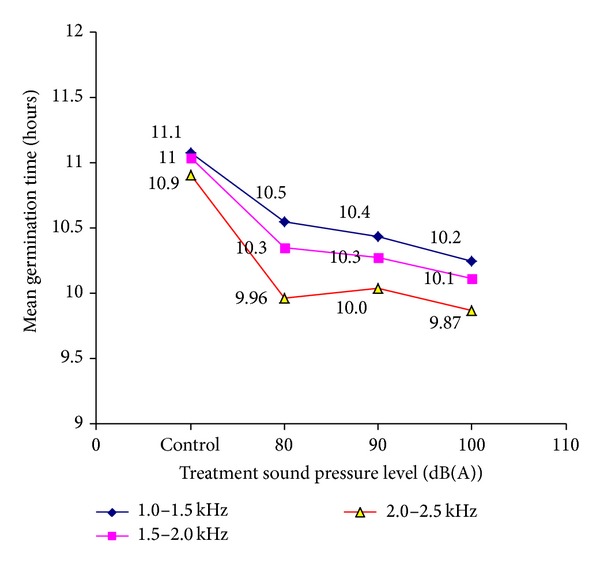
Mean germination time (MGT) of mung bean at various sound treatments.

Mean germination time was determined for all the treated groups and relative control groups. As shown in Figure 3, the germination period of mung bean was reduced after audible sound treatments. From Figure 3, it can be found that higher sound frequency band treated groups have shorter germination time, which means that there may be a correlation between the effects of degree of audible sound on the germination of mung bean and the sound frequency.

### 3.2. Audible Sound Effect on the Seedling Growth of Mung Bean

Growth data were measured at the 72nd hour after seeding. These data allow us to distinguish significant differences between fresh weight, stem length, and root length of mung bean seedlings subjected to audible sound with different swept frequency band (SFB) (1000–1500 Hz, 1500–2000 Hz, and 2000–2500 Hz) versus control.

**Figure 4:**
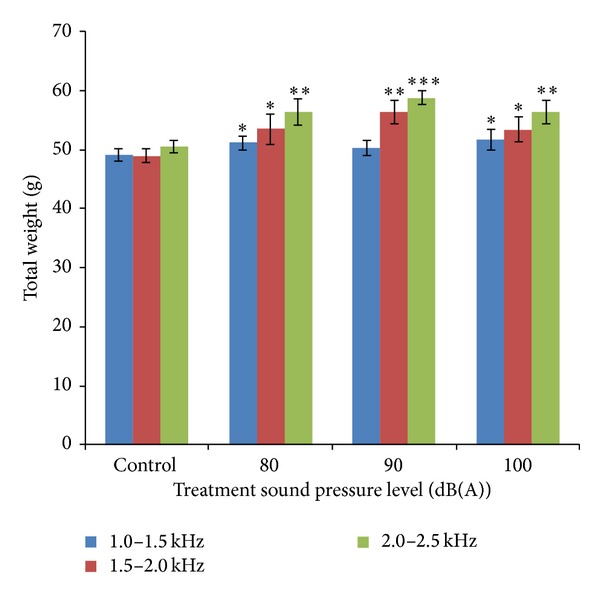
Total weight of mung beans measured at the 72nd hour at various sound treatments. Asterisks indicate significance: ***P < 0.001, **0.001 < *P* < 0.01, and *0.01 < *P* < 0.05. Vertical bars represent means ± SEM.

Figure 4 shows the total weight of mung beans measured at the 72nd hour at various sound treatments. Significant differences (0.01 < *P* < 0.05) were obtained when bean seedlings were exposed to the sound with SPL of 80 dB and 100 db. The greatest increases in weight were obtained for mung beans exposed to audible sound with SPL of 90 dB. Extremely significant differences are observed (*P* < 0.001) between mung bean seedlings continuously exposed to audible sound with swept frequency band of 2.0–2.5 kHz and the controls.

**Figure 5:**
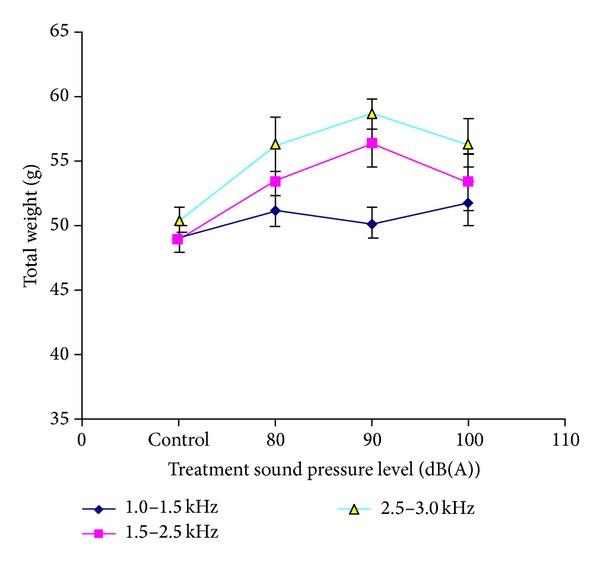
Comparison chart of total weight of mung beans measured at the 72nd hour at various sound treatments. Vertical bars represent means ± SEM.

Comparison chart of total weight of mung beans measured at the 72nd hour at various sound treatments is plotted in Figure 5. From the comparison chart, it can be found that the mung bean under treatments of sound with intensity of 90 dB and frequency around 2000 Hz significantly increases in fresh weight.

**Figure 6:**
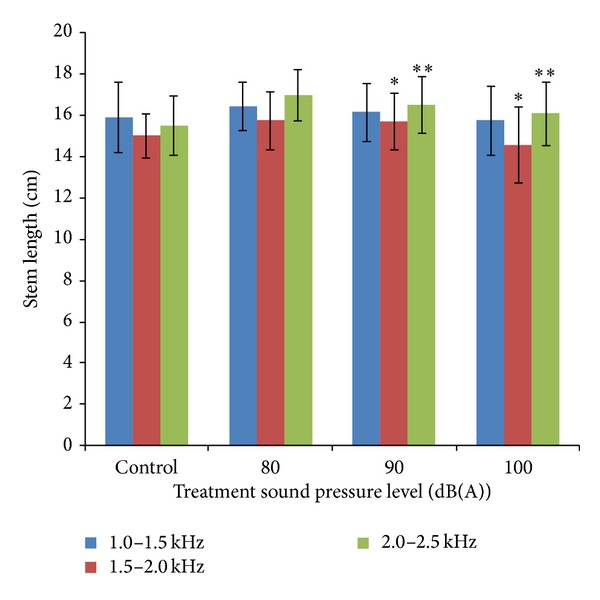
Stem length of mung beans measured at the 72nd hour at various sound treatments. Asterisks indicate significance: **0.001 < *P* < 0.01 and *0.01 < *P* < 0.05. Vertical bars represent means ± SEM.

Figure 6 shows stem length of mung beans measured at the 72nd hour at various sound treatments. As shown in Figure 6, there are no significant differences (*P* > 0.05) when bean seedlings were exposed to the sound with SPL of 80 dB, 90 dB, and 100 dB. Significant differences (0.01 < *P* < 0.05) were obtained when bean seedlings were exposed to the sound with SPL of 90 dB and 100 dB. Greatly significant differences (0.001 < *P* < 0.01) were presented when mung bean seedlings were continuously exposed to audible sound with SPL of 90 dB and 100 dB.

**Figure 7:**
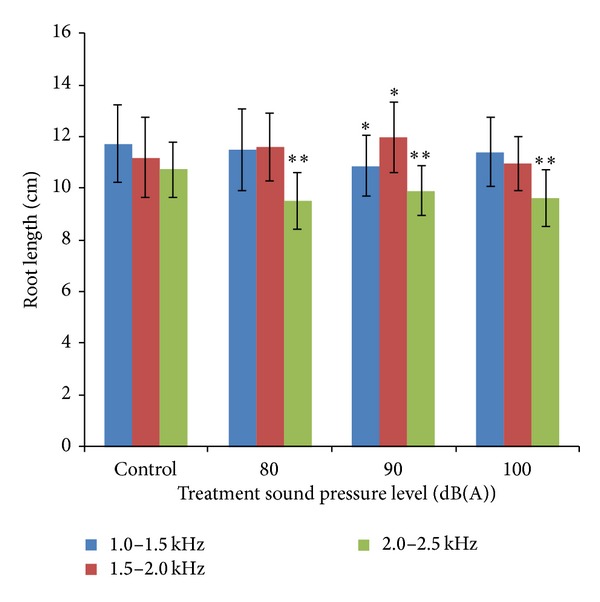
Root length of mung beans measured at the 72nd hour at various sound treatments. Asterisks indicate significance: **0.001 < *P* < 0.01 and *0.01 < *P* < 0.05. Vertical bars represent means ± SEM.

Figure 7 shows root length of mung beans measured at the 72nd hour at various sound treatments. There are no significant differences (*P* > 0.05) when bean seedlings were exposed to the sound with SPL of 80 dB and 100 dB; however, significant differences (0.01 < *P* < 0.05) were presented when mung bean seedlings were continuously exposed to audible sound with SPL of 90 dB. Significant differences (0.01 < *P* < 0.05) were also obtained only when bean seedlings were exposed to the sound with SPL of 90 dB. Greatly significant differences (0.001 < *P* < 0.01) were presented when mung bean seedlings were continuously exposed to audible sound with SPL of 80 dB, 90 dB, and 100 dB.

## 4. Discussion 

From this study, we found that the germination period of mung bean was reduced after audible sound treatments. Results are similar to those music effects on the germination of okra and zucchini seed reported by Creath and Schwartz [18]. The mechanisms are still unknown till now, but there is a hypothesis that the sound treatment of mung bean seeds in aqueous media can result in an advanced hydration process, which might be an explanation for the reduction in the germination period of mung bean.

Moreover, from our experiments, we found that significant effect of sound energy on germination time was obtained with swept frequency of 2.0–2.5 kHz and SPL 80, 90, and 100 combinations and with 1.5–2.0 kHz frequency and 90 SPL combinations. Improvement in stem and root lengths was seen only with higher sound frequency (2.0–2.5 kHz) and sound pressure levels of 80, 90, and 100 dB. In summary, the audible sound wave can reduce the germination period of mung bean and the mung bean under treatments of sound with intensity around 90 dB and frequency around 2000 Hz showed significant differences both in stem length and root length. Audible sound treatment can promote the growth of mung bean differently for distinct frequency and intensity. These results are interesting but there is no exact explanation for these results till now.

The possible mechanisms of audible sound effects on plants are not yet well known; however, several possible mechanisms have been proposed.

1. Audible sound stimulation might cause plants' leaf stomata to open, and then the plant will be able to absorb more spray fertilizer and dew. Moreover, the absorption efficiency of light energy might increase with audible sound, which would result in more light energy used for photochemical reaction and less superfluous excitation energy. Both sound energy and light energy could convert to and be stored as chemical energy, which would enhance the photosynthesis system [19]. Audible sound with certain sound frequencies might also help a plant breathe better and absorb more nutrients [20].
2. Plants might have a meridian system as in human or other animals [21, 22]. The frequencies of external sound stimulation along with the plant spontaneous sound frequency are in line and then the resonance occurs. Moreover, when sound wave energy reached leaves, part of sound energy vibrates leaves, other parts of sound energy reflect or diffract around those leaves, and part of sound energy affects the insects around plants.
3. There is a dissertation result which showed that audible sound with main frequency in nature sound collected from environment of wild plants (such as wild birds' chirm) has better effects on plant growth than other kinds of audible sound [15], which might imply that plants slowly accustomed to nature sounds of their environment of wild growth in the long-term evolution process. This explanation might offer us a good way to find best “sound frequencies and intensities” for different kinds of plant growth if it would prove to be right.

Therefore, further experiments should be conducted to confirm these possible mechanisms.

## 5. Conclusions

This study has demonstrated that a promising audible sound technique reduced the germination period of mung bean and improved the seedling growth of mung bean. The mechanisms of audible sound effects on plants are not well known yet. However, sound is effective for stimulating the germination and growth of mung bean suggesting that this technique has interesting possibilities in biophysics. In order to understand the effects of sound on plant growth and to develop models for application in agriculture production, more experimental studies need to be done and it is necessary to do collaboration work with agronomists, engineers, physicists, biologists, and so on toward establishing suitable mechanism of the biological effect on plants growth.

## Figures and Tables

**Table 1:** Sound treatments applied in this study.

Trial number	Sound pressure level (dB)	Sound frequency band (kHz)
1	Control (quiet)	1.0~1.5
	80	
	90	
	100	

2	Control (quiet)	1.5~2.0
	80	
	90	
	100	

3	Control (quiet)	2.0~2.5
	80	
	90	
	100	
